# Evaluation of a Rapid One-step Real-time PCR Method as a High-throughput Screening for Quantification of Hepatitis B Virus DNA in a Resource-limited Setting

**DOI:** 10.5005/jp-journals-10018-1121

**Published:** 2015-01-06

**Authors:** SM Rashed-Ul Islam, Munira Jahan, Shahina Tabassum

**Affiliations:** 1 Department of Virology, Bangabandhu Sheikh Mujib Medical University, Shahbag, Dhaka, Bangladesh

**Keywords:** HBV DNA viral load, One-step PCR, Two-step PCR, Resource limited settings.

## Abstract

Virological monitoring is the best predictor for the management of chronic hepatitis B virus (HBV) infections. Consequently, it is important to use the most efficient, rapid and cost-effective testing systems for HBV DNA quantification. The present study compared the performance characteristics of a one-step HBV polymerase chain reaction (PCR) *vs* the two-step HBV PCR method for quantification of HBV DNA from clinical samples. A total of 100 samples consisting of 85 randomly selected samples from patients with chronic hepatitis B (CHB) and 15 samples from apparently healthy individuals were enrolled in this study. Of the 85 CHB clinical samples tested, HBV DNA was detected from 81% samples by one-step PCR method with median HBV DNA viral load (VL) of 7.50 × 10^3^ lU/ml. In contrast, 72% samples were detected by the two-step PCR system with median HBV DNA of 3.71 × 10^3^ lU/ml. The one-step method showed strong linear correlation with two-step PCR method (r = 0.89; p < 0.0001). Both methods showed good agreement at Bland-Altman plot, with a mean difference of 0.61 log_10_ IU/ml and limits of agreement of -1.82 to 3.03 log_10_ IU/ml. The intra-assay and interassay coefficients of variation (CV%) of plasma samples (4-7 log_10_ IU/ml) for the one-step PCR method ranged between 0.33 to 0.59 and 0.28 to 0.48 respectively, thus demonstrating a high level of concordance between the two methods. Moreover, elimination of the DNA extraction step in the one-step PCR kit allowed time-efficient and significant labor and cost savings for the quantification of HBV DNA in a resource limited setting.

**How to cite this article:**

Rashed-Ul Islam SM, Jahan M, Tabassum S. Evaluation of a Rapid One-step Real-time PCR Method as a High-throughput Screening for Quantification of Hepatitis B Virus DNA in a Resource-limited Setting. Euroasian J Hepato-Gastroenterol 2015;5(1):11-15.

## INTRODUCTION

Viral hepatitis is the commonest liver disease in Bangladesh. Globally, over 350 million people are infected with hepatitis B virus (HBV), and around 1 million die annually due to the consequences of this infection.^1^ Bangladesh belongs to the intermediate prevalence region for HBV infection where carrier rates of varies from 7.5 to 10%.^2^ Studies from Bangladesh show that HBV is responsible for 31.25% cases of acute hepatitis, 76.3% cases of chronic hepatitis, 61.15% cases of cirrhosis of liver and 33.3% cases of hepatocellular carcinoma (HCC).^3-6^

Currently, available target amplification assays, such as the polymerase chain reaction (PCR), have a much lower limit of detection (as low as 100 copies/ml) and is becoming more widely available worldwide. They are preferable for the initial evaluation of patients and more importantly, for virological monitoring of both treated and untreated patients. Therefore, HBV DNA seems to be the best predictor in the management of HBV infection.^7-9^ As more HBV DNA quantitative assays become available, it is important to use an accurate, highly sensitive, real-time PCR having standardized target amplification technology for HBV DNA detection.^10^ In order to ensure comparability between the assays, HBV DNA levels should be universally reported in IU/ml that have been calibrated with the World Health Organization (WHO) international standard for HBV DNA.^11^ Simultaneously, the assay employed should equally quantify all HBV genotypes. As there are assay-to-assay variations in quantification of HBV DNA, the use of the same assay for a given patient is important in clinical practice to precisely monitor the antiviral efficacy of any given drug.^12^

DNA extraction efficiency varies from kit to kit and test to test, and the extraction process is not only time consuming but also tend to increase the risk of carryover contamination.^13^ Accordingly, direct PCR without DNA extraction for quantitative use has been reported in several studies.^14,15^ In this study, we analyzed the performance characteristics and comparability of two HBV DNA methods based on different technologies: a one-step (DNA extraction and amplification in single PCR tube) with a two-step (manual DNA extraction followed by DNA amplification) PCR method for quantification of HBV DNA. Performance characteristics, including analytical sensitivity, precision and reproducibility, were also studied.

## MATERIALS AND METHODS

The study included a total of 100 blood samples consisting of 85 randomly selected samples from chronic HBV infected patients who were referred from several health centers to the Department of Virology, Bangabandhu Sheikh Mujib Medical University (BSMMU), Dhaka, Bangladesh, for HBV DNA quantitative testing. Patients were recruited after taking their verbal informed consent. Another 15 samples from apparently healthy individuals; doctors, residents and laboratory personnel’s from the Department of Virology after assessing their HBsAg and anti-HBc (total) sero-negative status. Briefly, 4 ml of blood was collected in EDTA (ethylenediaminetetraacetic acid) containing tubes and plasma was separated and stored in multiple aliquots at -20°C until time of testing. All plasma samples were tested and quantified for HBV DNA using both the HBV PCR kits of different testing technologies; one-step (DNA extraction and amplification in single PCR tube) HBV PCR kit (Sansure, China) and the two-step (manual DNA extraction followed by DNA amplification) HBV PCR kit (AJ Roboscreen GmbH, Germany).

*One-step PCR method:* This method utilized 5 μl of nucleic acid lysis buffer to allow rapid lysis and release of HBV-DNA from plasma specimen within 0.2 ml PCR reaction tubes. Five microliter of test specimen along with 4 quantitative references (provided by the National Institute for Food and Drug Control, China) were added to the respective PCR reaction tubes, incubated for 10 minutes at room temperature, and 40 μl of reaction mixture was added. Each reaction mixture contained 38 μl of HBV PCR mixture (a pair of primer to target conserved sequence of HBV-DNA, specific probe, dNTPs, Mg^2+^, buffer solution), 2 μl of enzyme mixture (Hot start Taq enzyme, UNG enzyme) and 0.2 μl of positive internal control (cloning plasmid without HBV target sequence). HBV-DNA and the four quantitative references were detected in FAM channel (reporter: FAM, Quencher: None) and internal control was detected in VIC channel (reporter: VIC, Quencher: None). ROX was added as passive fluorescence dye to eliminate variations among different tubes and achieve more accurate quantification. HBV DNA quantification was performed using the following thermal cycling conditions: 50°C for 2 minutes, 94°C for 5 minutes followed by 45 cycle at 94°C for 15 seconds, 57°C for 30 seconds with total run time about 2 hours with the ABI 7300 real-time PCR system. Results were saved automatically upon completion of the reactions. Evaluation of precision and reproducibility of the one-step HBV PCR was performed with four plasma samples of different concentrations that were run in duplicates for two consecutive days considering two-step HBV PCR as a set method.

*Two-step HBV PCR method:* The HBV-DNA extraction procedure was performed with the INSTANT Virus DNA Kit (AJ Roboscreen GmbH, Germany) according to the manufacturer’s instructions. Briefly, 200 μl plasma was added into the tube containing 200 μl lysis solution along with 25 μl of proteinase K, mixed vigorously and incubated at 50°C for 15 minutes in a heating thermal block. Then, 400 μl of binding solution was added, passed through spin filter in a 2.0 ml receiver tube, and 500 μl of washing solution was added. After centrifuging at 12000 rpm for 1 minute, 650 μl of washing solution was added into new receiver tubes and centrifuged again. Then, 60 μl prewarmed elution buffer was added to yield extracted DNA which was stored at -20°C until DNA quantification. The extracted HBV DNA was amplified with RoboGene® HBV DNA Quantification Kit (AJ Roboscreen GmbH, Germany) according to the manufacturer’s instructions. Probes and primers of this kit were specific for a subsequence of the HBV-S gene encoding HBsAg which can amplify all eight HBV genotypes (A-H) with equal efficiency. Amplification of HBV DNA and standards were detected by the probes labeled with FAM/Green channel and internal control was detected in VIC/ Yellow channel. ROX was added as passive fluorescence dye to eliminate nonspecific amplification. HBV DNA amplification was performed with the ABI 7300 real-time PCR System using 5 μl of extracted DNA in a 25 μl of reaction mixture, containing 9.6 μl of PCR grade water, 2.5 μl of 10× PCR buffer, 5 μl of HBV/IC specific primer, probe and dNTP’s and 0.4 μl of Taq polymerase (5 U/ul). Thermal cycling conditions used were: 95°C for 4 minutes followed by 45 cycles at 57°C for 1 minute, 95°C for 30 seconds and 45°C for 30 seconds with total run time of approximately 3 hours. The pre-extracted standards were calibrated using a WHO calibrated reference HBV DNA preparation obtained from the German Federal Agency for Sera and Vaccines (PEI). Sample results were accepted only when the internal control was amplified. HBV DNA concentration was expressed in IU/ml.

*Linear dynamic ranges:* The linear dynamic ranges of the one-step HBV DNA quantification kit was 1 × 10^2^ to 1 × 10^11^ IU/ml, whereas it was 5 × 10^2^ to 5 × 10^9^ IU/ml for two-step HBV DNA quantification kit.

*Quality assessment of the laboratory work:* All the tests were performed maintaining proper specimen collection, separation, processing, and storage conditions as per laboratory criteria. Any cross contamination during the work were strictly controlled and retested when detected.

## STATISTICAL ANALYSIS

Sample results were log transformed for analysis. Spearmen’s correlation coefficient and linear regression analysis were performed to measure overall correlation between assays. Bland-Altman plots were used for analysis of agreement between the assays. All statistical analyses were performed with the SPSS 19.0 software package for Windows. A two-tailed p-value less than 0.05 was considered as statistically significant.

## RESULTS

Of the 85 samples from CHB patients, the one-step PCR system detected 69 (81°%) samples with HBV DNA level between 3.14 × 10^2^ IU/ml and 4.67 × 10^8^ IU/ml (median, 7.50 × 10^3^ IU/ml), whereas, the two-step PCR system detected 61 (72%) samples with VL ranging from 1.02 × 10^2^ to 3.13 × 10^9^ IU/ml (median, 3.71 × 10^3^ IU/ml). Of the total detected samples, 80% were detected by both one-step and two-step PCR systems, and were quantitated at various level with mean differences in quantification of 0.61 log_10_ IU/ml (one-step and two-step). All 15 apparently healthy individuals tested for HBV DNA by both the PCR kits had undetected VL. The features of both assays are described in Table 1. The intra-assay coefficients of variation (CV%) ranged from 0.33 to 0.59, while the inter-assay (CV%) ranged from 0.28 to 0.48 for the one-step PCR method (Table 2).

**Graph 1:**
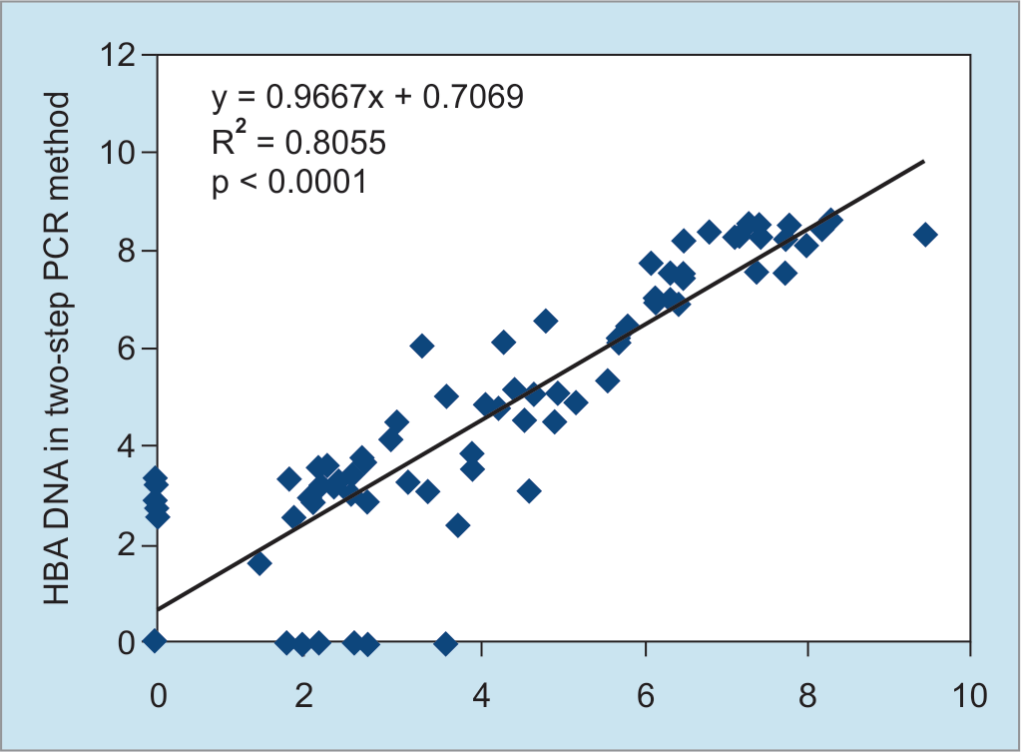
Linear regression analysis of HBV DNA levels (log_10_ I U/ml) between one-step and two-step PCR methods

**Graph 2:**
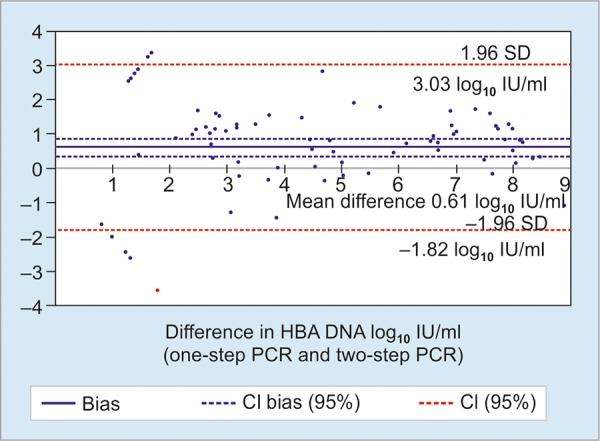
Bland-Altman analysis showing the limit of agreement in HBV DNA quantification between one-step and two-step PCR methods

Comparison of one-step and two-step PCR methods showed strong linear correlation between the assays (r = 0.89, 0.97; p < 0.0001) (Graph 1). These two PCR methods also showed good agreement at Bland-Altman plot, with mean difference of 0.61 log_10_ IU/ml (0.34 to 0.82 log_10_ IU/ml) and limits of agreement of -1.82 to 3.03 log_10_ IU/ml (Graph 2).

**Table 1:** **Table 1:** Features of one-step and two-step PCR methods

*Features*				*One-step PCR*		*Two-step PCR*	
Kit description				DNA extraction and amplification in single PCR tube		Manual DNA extraction followed by DNA amplification	
Lowest detection limit (IU/ml)				1 × 10^2^		5 × 10^2^	
Number of detected samples (n, %)				69 (81.2%)		61 (71.8%)	
Median VL (IU/ml)				7.50 × 10^3^		3.71 × 10^3^	
Category of VL (IU/ml)		<10^2^		16 (18.8%)		24 (28.2%)	
		101-10000		27 (31.8%)		22 (25.9%)	
		10001-999999		12 (14.1%)		15 (17.6%)	
		>10^6^		30 (35.3%)		24 (28.2%)	
PCR technology				Hot start Taq enzyme, UNG enzyme		Taq polymerase	
Log linear relationship		Slope		–3.44		–3.23	
		Intercept		46.83		42.31	
		Amplification efficiency (R^2^)		0.99		0.99	
Methods performance		DNA extraction		No		Yes	
		Run time (hours)		1:35		2:47	

**Table 2:** **Table 2:** Precision and reproducibility of one-step PCR method

*Expected VL* *(log_10_ IU/ml)*		*Interassay variations* *(log_10_ IU/ml)*								*Intra-assay variations* *(log_10_ IU/ml)*							
		*Run 1*		*Run 2*		*Mean*		*CV%*		*Replicates 1*		*Replicates 2*		*Mean*		*CV%*	
4		4.53		4.32		4.43		0.33		4.63		4.24		4.43		0.59	
5		5.62		5.31		5.46		0.48		5.58		5.31		5.44		0.42	
6		6.61		6.44		6.52		0.28		6.60		6.35		6.48		0.41	
7		7.67		7.41		7.54		0.41		7.60		7.39		7.50		0.33	

## DISCUSSION

Real-time PCR techniques have greatly improved the ability to quantify wide ranges of HBV DNA concentrations in patients with CHB infection and are the method of choice recommended by the American Association for the Study of Liver Diseases (AASLD).^16^ In this study, we verified the performance specifications of a European conformity (CE) and FDA-approved *in vitro* diagnostics (IVD)-licensed one-step HBV PCR method (Sansure, China) with CE-marked IVD-licensed two-step HBV PCR method (AJ Roboscreen GmbH, Germany) for quantification of clinical samples for HBV DNA. A variety of commercial assays with different dynamic detection ranges are available for quantitation of HBV DNA in diagnostic laboratories. In order to compare results of viral load generated from different manufacturers, it is essential to have validated, internationally acceptable standards.^17^ The one-step assay demonstrated excellent analytical sensitivity with a good dynamic range for detection of HBV DNA. Results of VL from the one-step PCR were compared with the two-step PCR validated with the WHO standard of HBV. Furthermore, one-step PCR method showed excellent correlation (r = 0.89, p < 0.0001) of the expected HBV DNA values with the two-step PCR.

Precision and reproducibility analysis is a basic requirement of a good quantitation assay, and the one-step PCR exhibited very low interassay and intra-assay variation in our study. The one-step PCR showed a maximum intra-assay variation of 0.59% at a 10^4^ IU/ml concentration and the assay was linear and reproducible between 4 and 7 log_10_ IU/ml. The type of samples used for accuracy of the results may also influence the determined performance characteristics of the assay. Therefore, fine tuning of this assay would make it a highly satisfactory HBV VL detection kit.

The main reason for difference between the two methods may be due to the difference in sample volumes, which were 200 μl for two-step and only 5 μl for one-step PCR. Besides, the final elution volume of 60 μl used in the two-step PCR made a substantial difference in the obtained DNA concentration, which was not required for one-step. The effect of sample volume on the sensitivity of HBV detection have been shown in various studies.^18,19^

Although, the one-step and two-step PCR methods employed different principles for sample processing and targeted different regions for amplification, the results for clinical samples correlated remarkably well. Moreover, avoiding the regular extraction procedure in the one-step HBV PCR limited the use of consumables plastic materials along with other necessary reagents to a great extent. This in turns greatly reduced the cost of the entire one-step PCR assay by nearly half the amount. The mean differences in quantification between assays were 0.61 log_10_ IU/ml and showed an adequate level of agreement. Therefore, both the PCR kits can be interchangeably used for therapeutic monitoring of CHB patients.

Our study demonstrated a high level of concordance between the one-step PCR utilizing direct plasma realtime PCR with the classical two-step real-time PCR for HBV DNA quantification. Eliminating the DNA extraction in the one-step PCR allowed considerable work and cost savings. In conclusion, the direct plasma one-step real-time PCR is a simple, economical, time-efficient and accurate method for quantification of plasma HBV DNA. Therefore, it may be used for high-throughput screening and evaluation of prognosis of CHB patients in resource-limited countries like Bangladesh.

The one-step PCR positive and two-step PCR negative or the vice versa results could not be analyzed due to variations in sample volume. Furthermore, precision and reproducibility were not vigorously checked due to budget and kit constrains.
